# The global epidemiology of Kawasaki disease: Review and future perspectives

**DOI:** 10.21542/gcsp.2017.20

**Published:** 2017-10-31

**Authors:** Ming-Tai Lin, Mei-Hwan Wu

**Affiliations:** Department of Pediatrics, National Taiwan University Hospital and College of Medicine, National Taiwan University, Taipei, Taiwan

## Abstract

Kawasaki disease (KD) is one of the most common childhood vasculitides and may lead to coronary arterial complications. KD has been reported in more than 60 countries over five continents. Previous publications have provided a comprehensive description of the epidemiologic features of KD including incidence, age of onset, seasonal trends, and rates of cardiac lesions. However, the interactions among the KD patients, time (seasons) and place have been less well studied. We review the current global epidemiology of KD and focus on the longitudinal changes in incidence, seasonality and response to intravenous immunoglobulin (IVIG) therapy.

## Introduction

**Figure 1.**
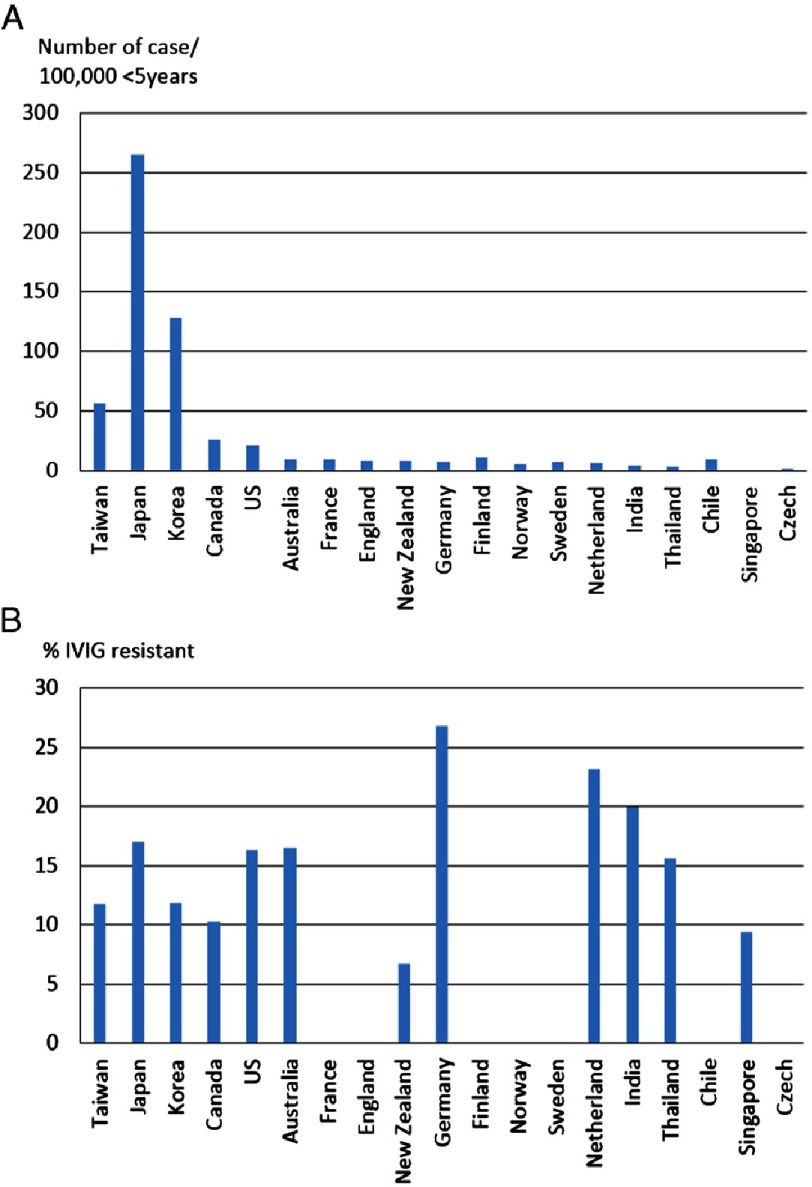
(A) Worldwide incidence of Kawasaki disease per 100,000 children younger than 5 years old; (B) Worldwide percentage of IVIG resistance in the children with Kawasaki disease.

Kawasaki disease (KD) has been described on every continent and cross all races and ethnicities^1–20^ [Figure 1]. Multifaceted epidemiologic studies describing incidence, distribution, seasonality and treatment of KD have provided important clues to the etiology and improved therapies for KD. Here we review the global epidemiology of KD and discuss potential implications.

It is important to understand that there are inherent limitations to the available epidemiologic data because, (1) the diagnosis of KD is clinically based, without a gold standard test, and (2) there is variation in methodologies to ascertain cases, including active surveillance reported by clinicians, use of immunoglobulin administration databases, center-based data registries, and national insurance (or hospitalization) records. Each of these data collection methods may result in some degree of both under- and over-reporting.

## KD in Asia

The incidence rates of KD in Asian countries, especially in Northeast Asia, are significantly higher than those in the USA and Europe^16,20^. Japan^4^, South Korea^5^ and Taiwan^6^ are the three Asian countries with reliable, robust nationwide KD incidence data that has documented a continuous increase in KD incidence. Because of the widespread knowledge about KD among medical practitioners in these countries, these rates reflect real increases in cases that are not affected by changes in physician awareness of KD.

### Japan

Japanese data are from nationwide epidemiologic surveys conducted every two years since 1970. The number of patients and incidence rates of KD have increased rapidly since the mid-1990s. The most recent published study was the 22nd survey^4^ conducted in 2013, which reported 26,691 KD patients (12,774 in 2011 and 13,917 in 2012). The annual incidence rates were 243.1 and 264.8 per 100,000 in children younger than 5 years in 2011 and 2012, respectively. This was the first time in Japan that the annual incidence of KD exceeded the rates observed in 1979, 1982 and 1986, when nationwide epidemics occurred. The number of patients with one or more siblings affected by KD was 1.5% and 0.89% of patients had at least one parent with a history of KD. Recurrence of KD occurred in 3.5% of cases and resistance to intravenous immunoglobulin (IVIG) was reported in 17.0% of cases. Only 4 KD patients died (4/26691, 0.014%) over the two-year period.

### South Korea

South Korea has conducted questionnaire-based nationwide surveys, similar to the Japanese surveys, from all hospitals with a pediatric residency program every three years since 1991. The incidence of KD in Korea is the second highest globally at 134.4 cases per 100,000 for children under 5 years of age^5^.

### Taiwan

Taiwanese data are based on the records of the National Health Insurance Program, which started in 1995 and covers >99% of the population (23 million people and approximately 5 million pediatric patients)^6^. Nearly every child in Taiwan receives complete medical services, therefore, the National Health Insurance Program can provide both longitudinal and cross-sectional data on KD.

From the longitudinal point of view, a national birth cohort (2000–2009) from Taiwan with complete postnatal data for more than 5 years (2000–2014) is appropriate for investigating the cumulative incidence of KD by the age of 5 years (2.78 per thousand) and the time trend with birth year (from 2.28 per thousand in 2000 to 3.67 per thousand in 2009)^6^. From the cross-sectional point of view, such a program can also provide an estimate of KD-associated complications, such as coronary artery sequelae^21^. Among the 23,349 KD patients (<40 years) identified from the 2000 to 2010 database, 1254 (5.37%) had various forms of coronary complications (861 males and 393 females).

## KD in Other Asian Countries

Incidence rates of KD have been reported across Asia using various methodologies, including China^22,23^, India^16^, Hong Kong^24^, Australia^9^ and New Zealand^10^. Most countries have a gradually increasing incidence of KD. The reasons for such increases are unclear and may be associated with a true increase or an increase in ascertainment due to increased awareness by health care providers and increased access to specialist care following economic growth and industrialization.

## KD in the Middle East

Limited patient series have been reported from countries in the Middle East, with the majority of the pediatric reports coming from Turkey and Iran^25–27^. Unfortunately, incidence data is not available from this region, perhaps because of the lack of census data for children <5 years of age. Of interest, a report from Egypt documents missed KD in an adult population presenting with myocardial infarction or acute coronary syndromes, suggesting that missed KD that is not diagnosed or treated may be prevalent in that country^28^.

## KD in North America

Epidemiologic surveys of US KD cases have relied mainly on passive national reporting to the Centers for Disease Control and Prevention, private insurance databases, or administrative databases such as the Pediatric Hospital Information Service. The occurrence incidence of KD in the US is estimated to be between 17.5 and 20.8 per 100,000 children <5 years^8^. Both data from the US and from Ontario, Canada^7,8^, showed prominent ethnic variation in the KD incidence rate, with a significantly higher rate among Pacific Islanders and Japanese Americans.

## KD in Latin America

Knowledge about the epidemiology of KD in Latin America is expected to improve with the formation of the Latin American Kawasaki Disease Network (REKAMLATINA), a multinational database from 20 Latin American countries organized in 2013^29–31^. This collaborative effort is a potential model for other regions of the globe. Data for cases are entered into a shared database and periodic analysis of cumulative data is planned. Chile has been at the forefront of epidemiological studies in Latin America^18^. The trend of an increasing number of KD diagnoses and hospitalizations was also found in Chile and thought to be due to improved awareness of the disease.

## KD in Europe

A recent review by Salo of KD epidemiology in Europe found that, in many European countries, the annual incidence increased during the 1990s and then stabilized^32^. Currently, the annual incidence of KD in Europe is about 5-10/100,000 children younger than 5 years^12–15,19,20^. The highest incidence of KD in Europe is in Ireland. Between 1996 and 2000, the incidence rate increased up to 15.2 per 100,000 children younger than 5 years^20^. The lowest rates are reported from three northern European countries with the following rates per 100,000 <5 years: Finland, 11.4; Norway, 5.4; and Sweden, 7.4^14^.

## Seasonality of KD

Several countries have reported distinct seasonality in KD, including Japan, Korea, Taiwan, USA, Canada and India^4–8,16^. Burns and colleagues^33^ analyzed the seasonal distribution of KD in the years 1970–2012 from 25 countries. They found a broad coherence in fluctuations in KD cases across the Northern Hemisphere extra-tropical latitudes, where KD case numbers were highest in January through March and 40% higher than in August through October. They later also proposed a hypothesis that tropospheric wind patterns from northerneastern China closely correlated with KD cases in Japan, Hawaii, and southern California^34,35^.

## Epidemiology of IVIG resistance

IVIG resistance is defined as persistent or recrudescent fever more than 36 hours after the end of initial IVIG infusion. Previous studies have shown IVIG resistance to occur in most populations worldwide^1^ (Figure 1B). Between 6.7% and 26.8% of KD patients from various cohorts were reported to be IVIG resistant and were at increased risk for coronary complications^4,5,7,9,10,13,15–17,36,37^. Interestingly, the percentage of IVIG resistance is not proportional to the incidence of KD. For example, the incidence of KD in children younger than 5 years was 264.8/100,000 and 55.9/100,000 in Japan and Taiwan, respectively^4,6^. However, the percentage of IVIG-resistance was 17.0%^4^ and 12.5%^38^ in Japan and Taiwan, respectively. In Germany, the KD incidence is 7.2/100,000 <5 years and the IVIG-resistance rate was high at 26.8%^13^. The reasons for such discrepancies are unclear. Delayed initiation of treatment, younger age, and brand of IVIG may all influence IVIG resistance. Genetic and immunologic approaches are being pursued to clarify the potential mechanisms of IVIG resistance.

## Future perspectives

Descriptive epidemiology has addressed three main issues, namely, person, time, and place of KD, and suggested hypotheses regarding the etiology of KD. However, the geospatial interactions have rarely been studied before. Recent studies of time series from 25 countries and composite atmospheric modeling were attempts focused on the interactions between time and place^33–35^. The concept of enrollment of a birth cohort to explore KD incidence in children born in different calendar years was another attempt to explore the interactions between time and cases. A global comprehensive registry and creation of KD networks with utilization of advanced computing and big data science may provide novel insights into the etiologies of KD.
